# Aptamer Bioinformatics

**DOI:** 10.3390/ijms18122516

**Published:** 2017-11-24

**Authors:** Andrew B. Kinghorn, Lewis A. Fraser, Shaolin Liang, Simon Chi-Chin Shiu, Julian A. Tanner

**Affiliations:** School of Biomedical Sciences, Li Ka Shing Faculty of Medicine, The University of Hong Kong, Pokfulam, Hong Kong SAR China; kinghorn@hku.hk (A.B.K.); lewis-fraser@hku.hk (L.A.F.); shaolin2@hku.hk (S.L.); simon156@hku.hk (S.C.-C.S.)

**Keywords:** aptamer, simulation, in silico selection, molecular dynamics, fragment based design, HTS

## Abstract

Aptamers are short nucleic acid sequences capable of specific, high-affinity molecular binding. They are isolated via SELEX (Systematic Evolution of Ligands by Exponential Enrichment), an evolutionary process that involves iterative rounds of selection and amplification before sequencing and aptamer characterization. As aptamers are genetic in nature, bioinformatic approaches have been used to improve both aptamers and their selection. This review will discuss the advancements made in several enclaves of aptamer bioinformatics, including simulation of aptamer selection, fragment-based aptamer design, patterning of libraries, identification of lead aptamers from high-throughput sequencing (HTS) data and in silico aptamer optimization.

## 1. Introduction

**Figure 1:**
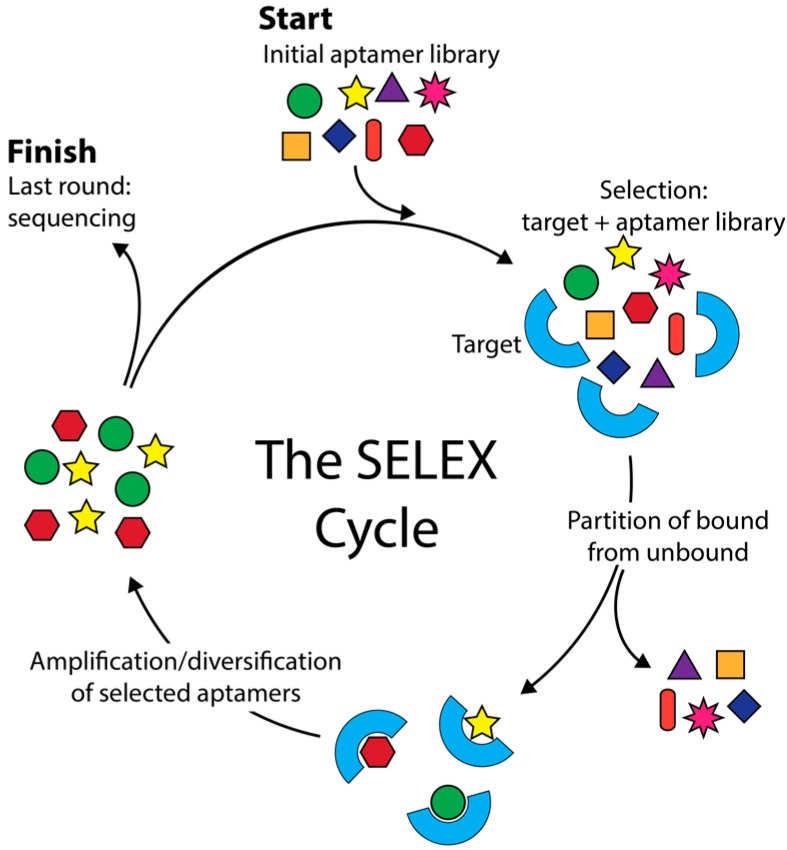
The SELEX (Systematic Evolution of Ligands by Exponential Enrichment) cycle. SELEX starts with a random nucleic acid aptamer library which is used to initiate the SELEX cycle (top arrow entering cycle). The library is incubated with the target and the target is washed to remove and discard unbound aptamers (right arrow exiting cycle) before the bound aptamers are eluted and amplified by PCR. The amplified sequences seed the next round of SELEX. Typically, around 12 SELEX cycles are performed before sequencing and aptamer characterization (left arrow exiting cycle).

Aptamers are short nucleic acid sequences capable of specific, high-affinity molecular binding [1,2]. Aptamers are isolated via SELEX (Systematic Evolution of Ligands by Exponential Enrichment) (Figure 1), an evolutionary process in which successive rounds of selection and amplification are used to enrich an aptamer library for high affinity aptamers. Aptamers are among the simplest of genetic entities, having both genotypic and phenotypic properties and being capable of heredity in an in vitro selection experiment. Their combinatorial complexity poses many questions and problems that are well suited to computational analysis. Many computational approaches have been applied to aptamers, bringing together different disciplines and technologies. This review encompasses a broad range of aptamer bioinformatics approaches including simulation of aptamer selection, aptamer selection by molecular dynamics, patterning of libraries, identification of lead aptamers from high-throughput sequencing (HTS) data, and in silico aptamer optimization. We aim to describe and contrast these methods so that aptamer scientists might make use of the diverse array of bioinformatics resources available. 

## 2. Simulation of Aptamer Selection

Aptamer selection is complex. Complexity is found in both the myriad of experimental parameters and the combinatorial complexity of nucleic acid libraries. McKeague et al. performed a statistical analysis of 492 SELEX experiments, investigating experimental parameters such as choice of target, selection template, pH, and temperature [3,4]. Specific parameters were shown to have a significant effect on the dissociation constant of the tightest binding aptamers [4]. This information is valuable to aptamer scientists, but is limited to routinely disclosed experimental parameters. Many useful experimental parameters are not routinely disclosed, such as mutation rate, target concentration per selection cycle, recombination techniques and the inclusion of novel unnatural bases. An exhaustive empirical analysis, involving SELEX with sequencing of every round, is limited further by the combinatorial complexity of nucleic acid libraries, which contain ~10^15^ sequences in an initial aptamer library pool [5]. Empirical analysis of anything close to this number of library members is simply not feasible. To investigate the experimental parameters of aptamer selection, simulation has been used. 

In 1991, SELEX was first simulated using a program named SELEXION (Systematic Evolution of Ligands by Exponential Enrichment with Integrated Optimization by Non-linear Analysis) [6]. SELEXION was first used to reconstruct bacteriophage T4 DNA polymerase gp43 SELEX experiments [2]. A library of eight random RNA bases underwent eight rounds of SELEX comprising ligand binding, partitioning, and amplification. Ligand binding was modeled using a kinetic mechanism between target-protein and all aptamer ligands that reach equilibrium, stated by Irvine at al. [6] as follows:

$$\left(Pf\right)+\left(RNAf_{i}\right)\;\;\begin{array}{c} \overset{k+i}{\to }\\ \underset{k-i}{\leftarrow } \end{array}\;\;\left(P:RNA_{i}\right)\;\;\;\;\;\;\;\;\;\;\;\;\;i=1,\ldots n,$$

where (*Pf)* was the free protein concentration, (*RNAf_i_*) was the free RNA species of *i* concentration, (*P: RNA_i_*) was the protein-RNA species *i* complex concentration, *k + i* was the rate constant for association of free protein and free RNA species *i*, *k − i* is the rate constant for dissociation of protein-RNA species *i* complexes, (*P: RNA_i_*) was the protein-RNA species *i* complex concentration, and *n* is the number of RNA sequences with a unique set of rate constants [6]. Partitioning efficiency for the reconstruction was set to 80% of bound aptamers and 0.1% of unbound aptamers. Amplification of partitioned aptamers involved reverse transcription to cDNA and PCR amplification before library generation using transcription. The experimental parameters of the gp43 selection [2] were reconstructed and underwent simulation as a proof of principle for SELEXION [6]. These simulations indicated that the equilibrium mechanism proposed above for SELEX was sufficient to explain the high levels of enrichment after just a few rounds observed in the laboratory experiments. Following the reconstruction simulation of the gp43 selection, several properties were investigated using SELEXION including predicted enrichment under different conditions, optimal protein concentration when dissociation constant (*K*_D_) estimates are known, near-optimum protein concentration with no estimate for *K*_D_, determination of sufficient protein concentration with no estimate for *K*_D_ or background, likelihood of SELEX success, and finally sequence representation in the random library pool [6]. SELEXION took a thorough approach to modeling ligand binding. However, a possible shortcoming would be the determination of aptamer properties such as *K*_D_. The binding affinities in terms of *K*_D_ were distributed without reference to aptamer sequence. For the reconstruction there were just five unique *K*_D_ values for all 65,536 unique aptamers in the initial library.

In 1998, Irvine et al.’s work [6] was extended and the program MultiSELEXION was coded to investigate SELEX against multiple targets [7]. MultiSELEXION allowed the investigation of problems arising from the use of contaminated protein preparations in SELEX, as well as analysis of complex target selections such as Cell-SELEX [8] and in vivo SELEX [9]. It was found that in most cases SELEX is capable of isolating differing ligands against the different targets in a heterogeneous mixture, irre SELEX spective of large variations in target concentrations or aptamer/target affinities. However, a low relative partitioning efficiency for a given target in a mixture gives a greatly reduced rate of selection of high-affinity aptamers [7].

Similarly to Irvine et al. [6] and Vant-Hull et al. [7], Chen et al. devised a SELEX simulation model that uses ligand binding based on equilibrium between target aptamer ligands and was applied to subtractive SELEX [10] as well as SELEX against a complex mixture [11]. This difference highlights and simulates selection pressures in SELEX experiments. Further similarities to Irvine et al. [6] and Vant-Hull et al. [7] included the binding affinities in terms of *K*_D_ being distributed without reference to aptamer sequence. Chen et al. used just 10 unique *K*_D_ values for all aptamers in the simulations [10].

Wang et al. developed a model that focused on the two SELEX parameters, target concentration and the effect of nonspecific binding [12]. The model represented ligand binding using equilibrium kinetics similarly to Irvine et al. [6] and Vant-Hull et al. [7]. Partitioning was modeled in two ways: without background binding, which was intended to mimic microfluidic selection; and with background binding, which was intended to mimic nitrocellulose filter-based separation. Aptamer binding properties were normally distributed [12] as hypothesized in the literature [13,14]. Wang et al. [12] found that “without background binding” conditions, an increasing amount of target decreases the selection efficiency. Under “with background binding” conditions, there is an optimum target concentration that increases with increasing background binding. Interestingly, under multiple selection rounds and “with background binding” condition the optimum target concentration for achieving maximum enrichment increases with each SELEX round. This is contrary to the generally accepted practice of reducing the target concentration as SELEX progresses. The reason for this trend of increasing optimum target concentration in successive SELEX rounds could be the modeling of background binders. The more target, the greater the number of specifically binding aptamers make it to the next round, therefore the higher the ratio of specific to nonspecific binders and the higher the average *K*_D_ value. One aspect that SELEX Wang et al.’s model and many other models do not take into account is the possibility of adaptation in the aptamer pool giving rise to aptamers with increasingly tighter *K*_D_ values. 

Spill et al. developed a model that simulates Capture-SELEX and includes non-covalent ligand–substrate immobilization [15]. Aptamer–target binding was represented using a hybrid approach whereby an equilibrium constant is combined with a stochastic probability model. Following partitioning, the amplification of selected aptamers was simulated. Of particular interest is that the initial library *K*_D_ distribution has a dramatic effect on the outcome of the simulation. Additionally, the impact of distribution noise and the downstream effects on the total target concentration were assessed. The use of a stochastic model and Monte Carlo simulation highlighted sensitivity of SELEX to stochastic variation. Twenty very tight binders are capable of outcompeting 10^15^ library members or can be totally lost.

Simulation of SELEX has given insight into how an aptamer scientist might optimize the SELEX protocol. SELEX has both vast complexity in terms of the number of sequences (typically around 10^15^), and informational complexity associated with each individual aptamer’s sequence, folding, and target binding. The aforementioned simulations have focused on representing the vast complexity of SELEX and neglected the informational complexity of individual aptamer sequences. All binding properties of aptamers are selected randomly or from a distribution with no relevance to the aptamer sequence. For particular questions about SELEX, including the role of adaptation and the occurrence of divergent and convergent evolution, a more thorough binding model is required.

Hoinka et al. coded a program to simulate the aptamer selection process called “AptaSim” [16]. AptaSim aimed at realistically recreating the selection process during SELEX with the intention of investigating the effect of error-prone PCR on aptamer selection. An initial library pool was generated using a first-order Markov Model, previously trained on early SELEX round selection data. The generated aptamer was randomly assigned a copy number and binding affinity within a predefined range. Iterative cycles of capture and amplification were then simulated where the capture probability is related to an aptamer’s copy number and binding affinity, and amplification is subject to a specified probability of mutation. The binding model used attributed aptamer affinities at random without relevance of sequence. Additionally, mutated versions of these aptamers retained the original’s attributed binding affinity. While AptaSim was an important step forward in simulating selection, enrichment and mutation copy number, AptaSim did not appropriately represent heritability or represent binding affinities correlated between related sequences, which is required for the study of SELEX as a genetic system. 

Oh et al. used a string matching function as a binding model to simulate aptamer selection [17]. All aptamers were given a target binding score based on their similarity to a given “optimal aptamer” sequence [17]. This model does include heritability and binding correlation between related sequences. As string matching is not computationally demanding, this approach can be used for very large library sizes, which is more representative of aptamer selection. The drawback of string matching is that only close-range epistasis is possible and by using a one “optimal aptamer” model, the landscape is cone-shaped and would not represent a true aptamer binding landscape.

Wedge et al. used Kauffman’s *NK* model [18] to represent ligand–target binding for the simulation of protein-directed evolution [19], a similar field to aptamer selection. The *NK* model is a robust mathematical model that serves as an objective function relating genotypic sequences to phenotypic fitnesses that make up a fitness landscape. Using the *NK* model, strings of informational digits of length *N* are attributed fitness values equal to the sum of each digits interaction with *K* other digits. In this way, epistatic and pleiotropic interactions can be modeled. The *NK* model has been used to describe many complex systems such as immunology [20], evolutionary biology [21], and economics [22]. The *NK* model has also been related to aptamers [23]. In Wedge et al.’s [23] work the ligand properties were determined using an *NK* model in which binary strings of length *N* = 100 were used with random epistatic interactions varying from *K* = 0 to 10. The initial library size was 40,000 and during each of the 10 selection rounds, 1 to 4000 of the tightest binding ligands were partitioned. Varying degrees of selection pressure (number of ligands selected each round), mutation rate, and crossover (recombination) were tested and it was found that optimal directed evolution (DE) parameters were strong selection pressure, a high mutation rate, and that crossover is only valuable when epistasis is low to moderate (*K* < 5). While these results are valuable to the field of protein-directed evolution, the simulation did not mimic properties specific to SELEX. 

The *NK* model can effectively represent the target binding of polymeric ligands such as proteins and aptamers. Besides the challenges for biological accuracy in representing base interactions within an aptamer, the classical *NK* model may have limitations in representing some aspects of biological systems. The *NK* model’s greatest utility is that epistasis can be tuned using the variable *K*. However, this epistasis is reasonably uniform throughout the sequence. To represent some biological systems, a higher amount of epistasis is desirable. As *K* increases the fitness landscape tends to become more rugged, to the point where it is too chaotic to allow adaptation, a phenomenon is referred to as the “complexity catastrophe” [24].

To overcome the “complexity catastrophe” and use the *NK* model to represent gene regulation, Altenberg [25] developed “selective genome growth” in 1995. Selective genome growth is an evolutionary approach that selects epistatic interaction in such a way to create a highly epistatic landscape that is smoother than classic *NK* landscapes with the same degree of epistatic interaction [25]. Altenberg’s selective genome growth *NK* landscape represents gene regulation very well. However, due to the increasing returns of the selection system, an extremely high pleiotropy is attributed to a handful of digits [26]. This highly aggregated pleiotropy is biologically appropriate and accurate for describing gene regulation. However, as each base in an aptamer has a relatively low number of interactions due to its spatial capacity, the highly aggregated pleiotropy is not biologically representative for base interactions within an aptamer.

**Figure 2:**
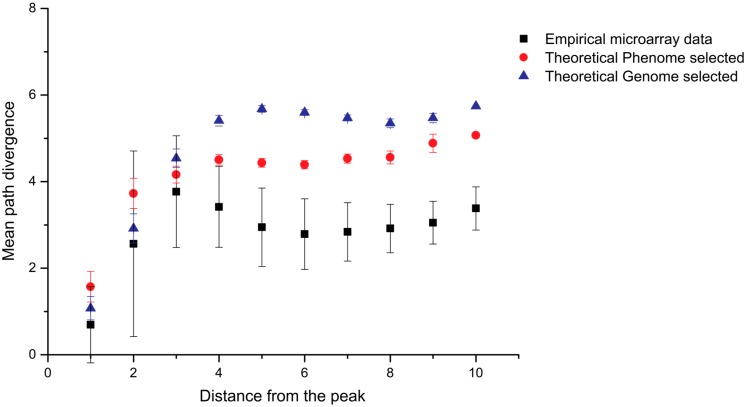
Comparison of novel phenome selected model to both genome selected and empirical aptamer microarray binding data. The mean path divergence analysis, a measure of landscape smoothness, shows that the novel phenome selected landscape is more similar to empirical microarray binding data than the previous genome selected landscape model. Figure adapted from Kinghorn et al. [26].

To overcome this problem Kinghorn and Tanner recently devised the method “selective phenome growth”, which generates fitness landscapes with low aggregated pleiotropy that more appropriately represent aptamer binding [26]. The selective phenome growth process involves phenotypic contributors being added to a genotype/phenotype interaction map sequentially in such a way as to increase the fitness of a selected “fit sequence”. In this way, a fitness landscape is built around the selected “fittest sequence”. The fitness landscapes obtained were compared to empirical aptamer microarray data and were shown to more accurately represent aptamer ligand binding than other theoretical models (Figure 2) [26]. The selective phenome growth model has not yet been utilized in the simulation of SELEX, only described and validated as a model that more accurately represents aptamer binding.

The no-free-lunch theorem states that all search algorithms perform exactly the same when averaged over all possible problems [27]. This infers that any elevated performance in one class of problem is exactly paid for in the performance of another class of problem. If there is discrepancy between a real-life system and a model used to describe it, for example an empirical SELEX experiment and a SELEX simulation, any elevated performance insight found using the simulation is exactly paid for in the performance of the real-life system. This illustrates the need for the simulation model to be as accurate as possible; otherwise optimizations will not translate to empirical SELEX experiments. The area least accurately modeled in SELEX simulations has been the aptamer binding model.

## 3. Aptamer Selection by Molecular Dynamics

Molecular dynamics have applications across biotechnology, including but not limited to protein studies, membrane transport, and drug discovery [28,29,30,31]. One particular application is to improve the efficacy of aptamer selections by computationally solving the three-dimensional structures of nucleic acids (NAs) and their targets, and simulating the physical forces involved in NA docking to a target. This is achieved by various N-body simulations that calculate the dynamic forces of the atoms and molecules of a NA within a binding site, in the form of a docking score. Docking scores can be used to identify sequences that bind to a target, defining a novel approach for aptamer discovery. Here, we will discuss studies wherein molecular dynamics has been used to enrich selection pools, optimize existing aptamers, and discover new aptamers.

### 3.1. Whole Aptamer Docking

For the purpose of this review we have divided in silico techniques into two categories: those that simulate the molecular dynamics of a whole aptamer and its target, and those that fragment an aptamer into discrete units to simulate binding interactions. We will discuss the literature that underpins in silico selections for whole aptamers. 

Computationally predicting secondary and tertiary structures of NAs and targets reveals the steric and energetic properties of each structure. These predictions allow researchers to modify their selection pools to have a broader range of three-dimensional structures and NAs with more favorable free energy [32,33,34], and provides essential information for molecular docking simulations [35]. Many protein-NA structures have been solved experimentally using NMR and X-ray crystallography, for which there are large but limited libraries in the Protein Data Bank (PDB). If the structure has not been solved experimentally, homology modeling webserver services exist for both proteins and NAs [36,37,38,39]. 

**Figure 3:**
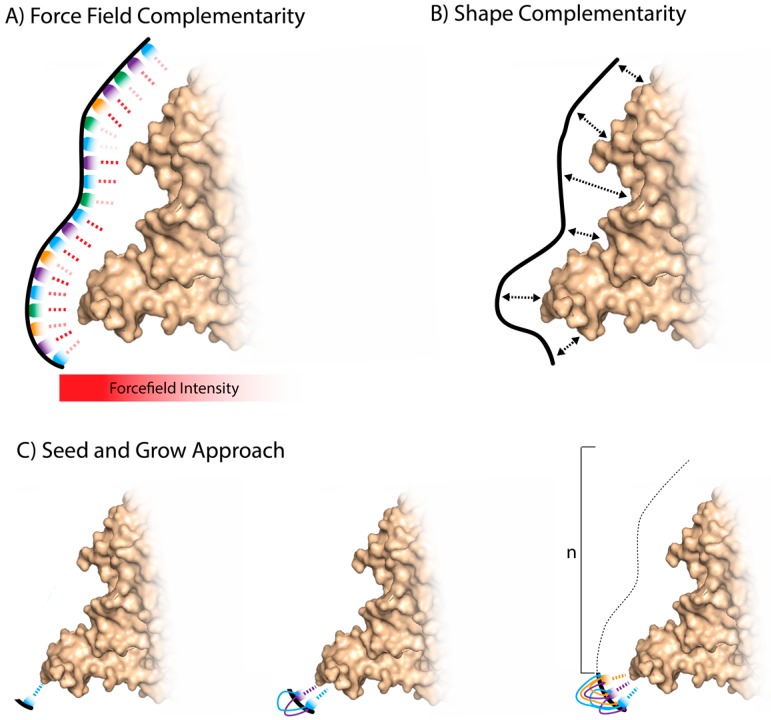
Simplified visualization of the molecular dynamics used in aptamer selections. (**A**) Force fields, the algorithmically calculated potential energies between atomic components of a nucleotide sequence and a target, are algorithmically calculated and represented here as a dashed red line. The differential opacity represents the variable favorability of each interaction, which is later translated into a docking score; (**B**) shape complementarity, a simplified representation of the nucleotide sequence interacting with a target based on spatial orientation; (**C**) the seed and grow approach, a fragment-based method. A single nucleotide is matched with a target and single nucleotides are added in sequence. A probability distribution is used to measure the interactions between the target and linked nucleotides. The target in this figure is derived from crystal structure PDB 3ZH2.

In simulating the docking between a target and an aptamer, several non-covalent interactions are assessed including ionic interactions, hydrogen bonds, van der Waal’s forces, hydrophobic interactions, base stacking interactions, and shape complementarity [35] (Figure 3A). Algorithms calculate the potential energy between interacting atomic components, known as force fields. For biological systems, the most frequently used MD force field simulations are CHARMM and AMBER [35]. Homology and modeling software for DNA–protein interactions is currently limited [35], as much of the software is based in analyzing protein–protein interactions. A coarse-grained force field has shown how dsDNA interacts with protein structures. Specific interactions are useful but limited in their scope and shape complementarity and internal DNA energy play an important role in simulating protein–DNA docking [40] (Figure 3B).

An initial attempt at in silico selection was proposed by Chushak and Stone. Computationally, they decreased RNA sequence search space in a selection pool by up to five orders of magnitude to enable conjugation of an enriched RNA selection pool to a microarray to improve high-throughput aptamer selections [34]. A three-step enrichment approach was used:

(1) Selection based on secondary structure—a set of criteria were used to identify and eliminate sequences with common simple structural motifs and high-energy unstable RNA sequences, both of which would be unlikely to form aptamers. 

(2) Selecting for conformational flexibility—a single RNA sequence can have a large range of three-dimensional conformations; the Rosetta RNA package [41] was used to generate these structures. Then the five lowest energy three-dimensional structures, and therefore those with the greatest conformational flexibility, were selected using the AMBER force field simulation and the generalized Born solvation model [42]. 

(3) Screening the RNA library with computational docking—a modified docking tool called DOVIS using Autodock v4 [43] was used to simulate interactions between all the generated RNA three-dimensional structures and small molecule targets. Docking was scored based on their calculated affinity for the targets. By selecting for the highest scoring sequences they effectively lowered the RNA pool size from ~2.5 × 10^8^ to 5 × 10^3^. Six known aptamer–ligand complexes were used to validate this approach. Native aptamers were found in within the top 5% of in silico selected structures. 

Confirming that molecular dynamic calculations align with experimental evidence provides further evidence that in silico approaches can complement aptamer selections. A software package that uses the CHARMM force field to analyze protein–protein interactions called Discovery Studio uses a docking simulation algorithm called ZDOCK [35,44]. ZDOCK was found to work effectively with short RNA–protein interactions [45] but was found to be ineffective when simulating longer RNA strands [46,47]. When combined with ZRANK, an algorithm that takes into account a range of attractive and repulsive forces, van der Waal’s forces, and desolvation, effective simulation of protein–long-strand RNA was achieved [35,48]. Having confirmed the efficacy of this software package in conjunction with aptamer–protein interactions, Chen’s research group mutated aptamers of angiopoiten-2 protein (Ang-2), a protein that regulates angiogenesis and is linked with the development and spread of cancer [49,50]. From the mutated strands, they selected three with high scores and tested them experimentally for binding with surface plasmon resonance (SPR). Based on binding affinity and SPR response, they claim one of these novel aptamers (Seq15_12_35, *K*_D_ 0.61) has improved binding when compared to a high-affinity Ang-2 aptamer (Seq1, *K*_D_ 1.39) found in the literature [48].

Selection can be a lengthy and costly process [51], especially when targeting human proteins for which native proteins may be expensive or commercially unavailable [52]. To lower the cost of selection, it is common to select an aptamer towards a recombinant or non-human version of the equivalent human protein [53]. There is an increased risk that the difference in homology between the native protein and the recombinant/non-human protein will result in selecting for an aptamer that will not bind to the native protein. This is the case for immune-checkpoint blockade receptor TIM3 [54], for which aptamers selected for murine binding aptamers lacked cross-reactivity with the human form. Based on the murine aptamer, Rabal et al. used a three-step bioinformatics process similar to those already discussed, but coupled cluster analysis with their chosen 3D docking algorithm, 3DRPC [55]. Clustering algorithm GROMACS [56] revealed highly populated clusters focused around specific binding sites. In four out of five cases, combining clustering with docking simulation revealed a binding mode and site that were not identified by docking simulation alone. They were able to show the scope of in silico aptamer–protein analysis by identifying a plausible binding site on murine TIM3 and aptamer binding mode that explains the lack of cross-reactivity in murine over human TIM-3 [54]. 

### 3.2. Fragment-Based Aptamer Design and Docking

Whole aptamer selections require massive computational resources and three-dimensional structures of both nucleotides and target. A fragment-based approach has been argued to simplify the process of in silico aptamer generation [57]. Tseng et al. presented a three-step approach in which they only require structural information of the target, known as the entropic fragment-based approach (EFBA) [57]. They first determined the probability distribution of the first nucleotide binding to the target. They then sequentially added nucleotides to the first, taking into account the probability distributions of the added nucleotide to its neighbors and the target. Finally, they determined a cutoff length based on an entropic criterion (information theory entropy). Once the target–NA complex was saturated and the interactions of the complex were at a global minimum irrespective of nucleotide additions, the sequence was selected [57] (Figure 3C). They developed the in silico “seed and grow” method by selecting two aptamers. One aptamer bound to the target phosphatidylserine (PS), which previously had no reported aptamer [57]. They have since continued their investigation on the PS binding aptamer with more computational and experimental detail with a view to translation for practical use [58]. 

## 4. Patterning of Libraries

In a SELEX experiment, typically nanomoles of aptamer library or approximately 10^15^ molecules are used. The typical length of a nucleic acid in a library is around 40 bases (total sequence space 4^40^), so less than one in 1.2 billionth of the sequence space is covered. Aptamers generally require secondary structure to bind their targets, therefore increasing the occurrence of secondary structure in the library should enhance the success rate when selecting for an aptamer. Here, we will outline several approaches that have been taken to pattern aptamer libraries with secondary structures.

RNA aptamer and ribozyme selection analysis has shown that the presence of distinctive secondary structures, such as a stem–loop, enhances the binding affinity [32,59,60]. Such analysis began by observing the nucleotide distributions. Schultes et al. [61] found that functional RNAs have a tendency to have more purine than pyrimidine. This correlation was studied by functional class and phylogenetic domain. It was found that the G+A and G+U content in archaea, bacteria, and eukaryote functional single-stranded RNA showed a similar positive bias and that the bias was inversely proportional to the sequence length [61]. Knight et al. performed a comparative analysis of distantly related and unrelated sequences using simplex to study all possible composition vectors (G+A, G+U and G+C) of isoleucine aptamer and hammerhead ribozyme [62]. A library size of 6.23 × 10^9^ sequences containing 25% U, 15% C, 20% A, and 40% G could maximize the probability of identifying both motifs (99%). This study demonstrated that adjustment of base composition could be used to lower the total number of candidates in a SELEX (Systematic Evolution of Ligands by Exponential Enrichment) experiment.

Computational methods use sequence information to pattern initial libraries, which results in the evolution of more complex structures. For DNA aptamer selection, Ruff et al. patterned their initial library pool with alternating purine and pyrimidine, which was found to increase the formation of stem–loop structures that bind to streptavidin, immunoglobulin E (IgE), and vascular endothelial growth factor (VEGF) [63]. By sequencing pools from each selection round, they compared the selection efficiency for both random and patterned libraries. The patterned library was significantly enriched relative to the unpatterned library at the 10th round. For IgE, after adding restriction endonuclease to digest the tagged sequences, it was found that the enrichment was further significantly increased. The use of a patterned library in SELEX was able to select specific binders for all three molecules with affinity at nanomolar levels better than those selected from random libraries (streptavidin: *K*_D_ = 105 nM, IgE: *K*_D_ = 26 nM, VEGF: *K*_D_ = 45 nM). These results showed that the use of a patterned library could increase the proportion of active aptamer, speed of selection, and affinity of the resultant aptamers [63].

This alternating purine and pyrimidine patterning strategy was enhanced when Martin et al. used a novel computational method to increase the structural complexity of a DNA library (Table 1) [64]. This patterning method reduced the size of the library, allowing the integration of the entire library onto a microarray, for the identification of a thrombin binding aptamer. The initial library was designed with UNAFold software using three major constraints. First, the first nucleotide of the aptamer must pair with the final one. Second, the number of unpaired bases must fluctuate between 10 and 30 for the 50-nucleotide strands. Third, there must be at least two stretches of unpaired nucleotides. This limited the total number of candidates in the library to 50,000 sequences. The selection results showed that the first six to eight bases of the top 15 sequences resembled thrombin binding aptamer and binding was also specific to thrombin. This demonstrated the effectiveness of using a patterned library on a microarray to select for aptamers. 

The 1963 discovery of Hoogsteen base-pairing explains the formation of triplex and quadruplex structures [65,66]. The G-quadruplex structure now holds significant interest due to applications in therapeutics [67,68] and diagnosis [69,70]. A G-quadruplex usually consists of four guanine tracks and a few tetrads [71]. The structural features include a broad surface of π-orbitals above and below the quadruplex that allow hydrophobic binding to targets such as nucleolin [72], hemin [73,74], and light-up fluorogens [75,76,77]. 

**Figure 4:**
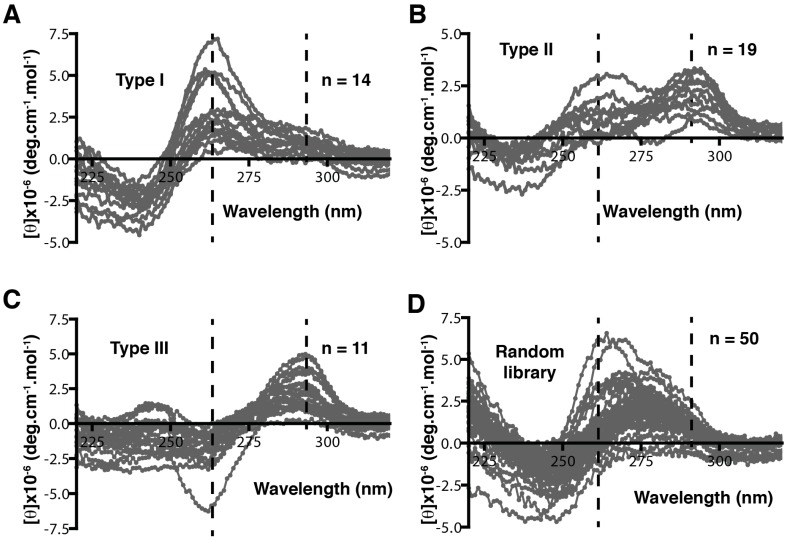
Circular dichroism (CD) characteristics of different quadruplexes and random library. (**A**) Parallel quadruplex shows positive peaks at 265 and 295 nm; (**B**) antiparallel quadruplex with glycosidic bond angles of the same orientation shows bimodal spectra as positive peaks at 265 nm and 295 nm of the same intensity; (**C**) antiparallel quadruplex with glycosidic bond angles of opposite orientation—the CD shows a negative peak at 265 nm and a positive peak at 295 nm; (**D**) random library shows a peak at 280 nm but no peaks at 265 nm and 295 nm, indicating the absence of a quadruplex. Figure adapted from McManus and Li [78].

The binding capacity of G-quadruplex structures was exploited by McManus and Li, who integrated patterning into DNA libraries to select aptamers with quadruplex structures. They patterned the library with the following methods: inclusion of four G-tracts in the library while leaving the rest to be random; simplifying the complicated three-layer G-quadruplex into a two-layered structure; and four G_2_ tracts were designed with three domains of random sequences for loop formation [71,78]. The authors first investigated the effective loop length for the folding of a quadruplex by adding three to seven thymidines between the G-tracts and characterizing by circular dichroism (CD). The CD characteristics of different quadruplex configurations are shown in Table 2. When compared to a completely random library, the G_2_ tract library showed peaks at 265, 280, and 295 nm, indicative of G-quadruplex structures (Figure 4), whilst the random library only showed a single peak at 280 nm. The authors also investigated the melting temperatures of individual libraries with different loop lengths at 295 nm. Libraries with a loop length of 3–6 were suitable for the selection of a single-stranded aptamer as their melting temperature did not change at increased concentrations. However, the melting temperature of a library with a loop length of seven increased with concentration. This indicated that it formed multimolecular quadruplexes because the interaction between large loops of different strands has a more regular structure and is not ideal for SELEX, requiring certain structural flexibility in the library. This pioneering work shows how optimizing DNA library parameters can maximize the possibility of selecting active binders.

In a SELEX experiment, the random library provides low sequence space coverage and low structure space representation. Using stem–loop structures and/or patterned libraries can increase sequence space coverage and improve space representation. Although it is difficult to pinpoint the best method to pattern a library, it is clear that patterned libraries can enhance aptamer selection by reducing the time taken to select an aptamer, increasing the success rate of a selection, and improving the binding affinity of isolated aptamers [63].

## 5. In Silico Aptamer Identification from High-Throughput Sequencing (HTS) Data

### 5.1. The Trend of Using HTS for Improving SELEX

Initially developed for the purpose of tackling the increasing complexity of whole genome sequencing, HTS technologies have continued to evolve and change the landscape in many fields of biomedical research over the last 10 years [79]. Since the development of the first commercialized 454 sequencer, companies such as Illumina, Ion Torrent, and Oxford Nanopore technologies are all increasing sequencer capacity and reducing cost [80]. In the area of aptamer research, deep, high-throughput, and in-parallel DNA sequencing technologies allow the analysis of millions of sequences found in each round of aptamer selection, and thus open a new avenue for identification and optimization of aptamers [81]. HTS data obtained from each round of the selection can not only be used to monitor the dynamic sequence change of aptamer selection to identify the best-performing sequences in early rounds [82,83,84], but also as a tool for aptamer scientists to further investigate the enrichment principles of SELEX process such as selection efficiency [85], aptamer–target interactions [86], and mutation landscape [16].

Some of the earliest works applying HTS for identification of aptamers were performed by Schroeder’s group in 2010. In the study, they applied a genomic RNA library—overlapping sequences constructed via PCR from the *E. coli* genome—to select against an RNA binding protein named Hfq. 454 sequencing was used to obtain sequence data for two of the last round libraries from the genomic selection and, for comparison purposes, the rounds of another selection that omitted the target binding step, to monitor the amplification variants of the genomic SELEX. By analyzing the HTS data, they successfully identified genomic RNA aptamers and discovered that these aptamers are predominant in the antisense transcripts [87,88]. In the same year, Soh’s group pioneered DNA aptamer quantitative selection by applying microfluidic and HTS technologies. They performed three rounds of microfluidic device-assisted selection against platelet-derived growth factor BB (PDGF-BB) and sequenced each round of the selection via high-throughput sequencing. More than 1.7 × 10^7^ sequences from each round of selection were obtained and the enrichment trajectory across different rounds was tracked by analyzing the HTS data. Comparing the sequences obtained from different rounds, they discovered the sequence with the highest affinity did not have the highest copy number in the last round [89]. Schultze et al. [90] confirmed this finding when they discovered that the library convergence in SELEX led to high-performance sequences being outcompeted by weaker-performing sequences that amplify more efficiently during PCR. The best binders tend to enrich rapidly in the very early rounds of selection [90]. Spiga et al. [91] performed HTS and SPR to monitor the binding affinity change and aptamer enrichment for tobramycin selection. They also discovered the most enriched and best binding sequences are visible even after two selection rounds [91]. As the cost of HTS continues to decrease [92], more researchers use it for characterizing multiple selection round libraries to ensure the quality of selected candidates [84,93]. Using the HTS dynamic monitoring method, researchers successfully identified high binding aptamers both for proteins [94] and small molecule targets [91]. 

### 5.2. Benchmark Toolkit for HTS SELEX Analysis 

Besides the cost, one of the major hindrances in early years to generalize HTS methods for aptamer identification was the difficulty of processing large amounts of sequence data. However, multiple open-source/paid bioinformatics tools have been developed specifically for aptamer scientists. The initial step in processing HTS data from a sequenced SELEX pool is to remove the adapter, barcode, or constant region from the sequences. After this pre-processing, a tool to count the sequence frequency is required. Previously, aptamer research groups used genomic informatics software packages such as Tallymer [95,96] or RazerS [90,97], or designed in-house programs to fulfill the counting requirement [89,98]. 

Galaxy Project is a platform that provides fundamental bioinformatic tools for bench scientists who may not have a background in bioinformatics. Thiel et al. recently developed workflows based on this Galaxy Project for handling HTS SELEX data to perform pre-processing steps [99,100]. This tool also allows researchers to remove adapter/barcode/primer regions from sequences; identify and remove sequences with mismatches within the primer region; set a variable region length cutoff; and count the number of duplicate reads. Another benefit of the Galaxy workflow is it is “ready to use” and “easy to access” for an open-source, web-based platform. However, the Galaxy web service does not currently provide analysis for motif-based clustering as the platform was designed for general genomic projects. 

An easy-to-use, aptamer-specific bioinformatics tool to address the clustering based on primary sequence is FASTAptamer [101]. FASTAptamer consists of a library of modular Perl scripts and is compatible across UNIX-like systems (or a Windows system with a Perl interpreter installed). Count, compare, cluster, enrich, and search are the five major script modules. By using these modules, users can count, normalize, and rank the sequence reads in a FASTQ file and group these sequences into families based on Levenshtein distance, as well as determine the SELEX enrichment across multiple selection rounds [101]. PATTERNITY-seq, developed by Ducongé’s group [102], is another package that uses sequence pattern clustering based on Levenshtein distance. They validated this approach by re-analyzing the data from a previously published cell-SELEX against Annexin-A2 [103].

**Figure 5:**
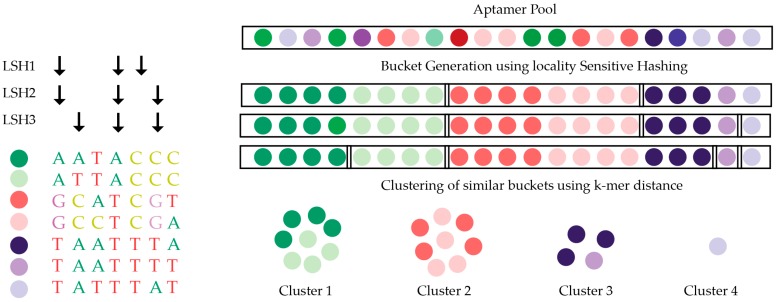
Illustration of the AptaCluster algorithm. Each colored sphere represents an individual sequence in the library and the similar colors represent related sequence. AptaCluster clusters the library pool into different sets of similar sequences based on locality sensitive hashing (LSH). The black arrows represent the user-defined number of nucleotide positions, which are sampled to generate input for the hash function. Figure adapted from Honika [16].

AptaCluster [104] is similar to FASTAptamer but based on the local sensitive hashing (LSH) method, which is capable of comparing sequences with a reduced number of dimensions (Figure 5). Iterative rounds of comparison within groups of aptamers are used to cluster aptamer sequences. By using such a method, the computational time required for AptaCluster is less than FASTAptamer. However, this method cannot be applied as a sequence pool containing various sizes. FASTAptamer and AptaCluster are purely text-based tools, whereas a program called AptaGUI that can be used alongside AptaCluster includes a graphical user interface (GUI) for the dynamic visualization of HTS SELEX data [105]. An alternative to AptaGUI is the paid platform COMPAS, developed by AptaIT GmbH. This also contains GUI for the navigation of the HTS data, but many of the operational details are proprietary and the company program is only available in conjunction with the purchase of their selection service.

### 5.3. Structure Motif Clustering-Based Tools

Most of the previously mentioned bioinformatics tools do not include functions for prediction and clustering of HTS data for structure-based methods. Furthermore, they do not allow elucidation of complex motifs and important pre-processing steps for initial analysis of the data generated during SELEX. 

Structure prediction programs such as Mfold [106] have been used to analyze low-throughput sequence data. Mfold predicts the secondary structure of single-stranded nucleic acids by energy minimization. Even though “bulk” servers of Mfold can analyze hundreds of sequences at once, it is, however, difficult to handle structure prediction on the HTS scale. 

A recently developed platform for structure motif clustering is AptaTrace [107]. Based on the secondary structure prediction from SFOLD [108,109], AptaTrace applies this information into all of the sequences input to the program. This allows for the prediction of a specific structure for each k-mer in each selection round and ranking by predicted significant structural enrichment. APTANI is a similar program, able to cluster sequence motifs based on secondary structure prediction. It uses RNAsubopt from the Vienna RNA package [110] and predicts using sub-structures, apical loops, bulge loops, and intra-strand loops. This method was validated using a SELEX against IL4Ra. Using APTANI, an aptamer was identified in one round, which previously required five rounds. 

The speed of HTS technology adoption has motivated the development of particular tools to assist HTS-based SELEX and identify better aptamer candidates (Table 2). Even though many approaches still lack multiple validations, using HTS to replace conventional sequencing methods for aptamer development is the trend. Recent progress in this field shows the potential for developing an all-in-one bioinformatics tools for aptamer researchers.

## 6. In Silico Aptamer Optimization

Aptamers have been isolated with both high affinity and high specificity for binding to their selected targets [5,111]. SELEX is an efficient method of isolating aptamers; however, following selection an aptamer scientist must always ask “Have I isolated the best possible aptamer sequence?” The library used for SELEX generally has a random region of around 40 bases [4], and typically only a few nanomoles can be used for the initial selection round. This represents a sequence space coverage of one in 80 billion. From this incredibly small sequence space coverage, it is unlikely that one will select the single best aptamer sequence. Sequence adaptation via mutation may account for some sequence space searching; however, as selection for SELEX is relatively low resolution [112], it is difficult to resolve the very best aptamer sequence.

Bioinformatic approaches have been used to improve the affinity of aptamers. As highlighted earlier, due to low selection pressure classical SELEX is unlikely to resolve the very best aptamer sequences. Therefore, each individual aptamer generated using a bioinformatics approach must be individually assayed for binding affinity, which can be labor-intensive and time-consuming. DNA microarrays consist of many features or spots on a glass slide, each feature containing many copies of a unique DNA sequence. This high-throughput technology allows for simultaneous assay of many aptamer sequences via incubation with fluorescent target.

In 2007, Katilius et al. used DNA microarrays to optimize and explore the surrounding sequence space of an aptamer against immunoglobulin E (IgE) [113]. Variations of the aptamer sequence with single, double and a selection of triple point mutations were synthesized onto a DNA microarray and assayed with Alexa Fluor 647 labeled IgE. This mutational analysis highlighted the conserved and unconserved base positions in the aptamer sequence. One aptamer variant showed mild affinity improvement (*K*_D_ = 4.1 nM) when compared to the original aptamer sequence (*K*_D_ = 4.7 nM) [113]. 

Platt et al. analyzed the sequence activity relationship of a set of G-quadruplex thrombin binding aptamers using DNA microarray technology [114]. The combinatorial landscape was probed via two methods. The first method investigated two internal loops of the G-quadruplex with 2-3 base random regions (GGGGAGTAGG(X_2–3_)GGTGTTGG(X_2–3_)GGGGCTCCCC, where X denotes the bases varied). The second method investigated the hairpin in which the G-quadruplex is nested within a section using pseudo-random variants ((X_8_)GGTT(X_2–4_)GGTTGGGG(X_6_), where X denotes the bases varied). Despite this search through sequence space, the tightest binding novel aptamer (*K*_D_ = 28 nM) had a lower affinity than the original ThB aptamer (*K*_D_ = 26 nM) [114].

Knight et al. combined a DNA aptamer microarray assay with in silico closed-loop aptameric directed evolution (CLADE) to select for aptamers against the natively fluorescent target allophycocyanin (APC) [115]. Five hundred control aptamer and 5500 test pool aptamers of 30 nucleotides were synthesized onto a DNA microarray for each round. The initial test pool for the first round was randomly generated. The test pool aptamers were assayed on the microarray for APC binding and ranked according to binding score. The top four aptamers were then subjected to point mutations and insertion–deletion events to give rise to a new 5500 test pool for synthesis onto a DNA microarray and use in the next round of selection. Nine rounds of CLADE were performed and the resulting aptamers characterized and phylogenetically analyzed. The CLADE strategy was successful with the tightest binding aptamer had a SPR determined *K*_D_ value of around 2 nM [115]. Although high-affinity aptamers were isolated, the cost of nine microarrays would be much greater than the cost of an average SELEX experiment.

Expanding upon this work, Rowe et al. used the CLADE approach and tested the three diversification systems: mutation, recombination, and statistical binding prediction [116]. Over five CLADE selection rounds, aptamers were evolved to bind to glucose-6-phosphate dehydrogenase. The tightest binding aptamer was isolated using the recombination diversification system and had a *K*_D_ of 245 nM.

In 2012, Nonaka et al. used an in silico system to improve the affinity of the VEap121 aptamer against VEGF [117]. Interestingly, this study did not use DNA microarrays but instead used SPR to assay every individual aptamer. Three rounds of improvement were performed. Each round consisted of adaptation, SPR determination of *K*_D_ value, and selection of the five tightest binding aptamers to seed the next round. For the first generation, 10 mutants of VEap121 were generated, each with several mutations, where the guanine bases were conserved to retain the G-quadruplex structure. For the second generation, the five tightest binding aptamers from G1 as determined by SPR were replicated relative to their binding affinity to yield 20 sequences. These were then randomly paired to undergo single-point crossover and two single-base mutations, randomly introduced. For the third generation, the five tightest binding aptamers from both G1 and G2 as determined by SPR were crossed with VEap121 at a random point and two single-point mutations were randomly introduced. This process was repeated three times in order to produce the third generation of 20 sequences. This process produced four aptamers with a tighter binding (*K*_D_ = 0.3, 1.5, 1.7 and 2.4 nM) than the original VEap121 aptamer (*K*_D_ = 4.7 nM) [117].

**Figure 6:**
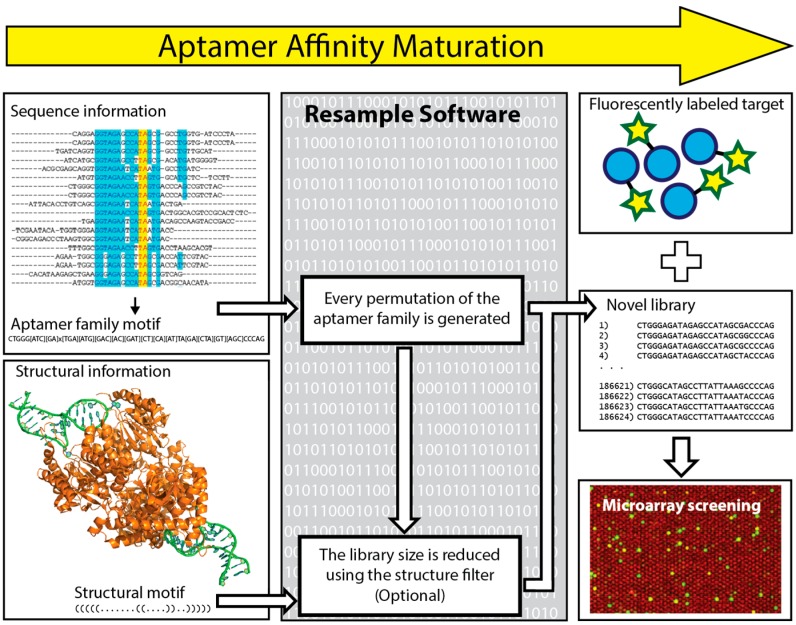
The aptamer affinity maturation process. The yellow arrow designates the aptamer affinity maturation process, in which data from a SELEX experiment is utilized by bioinformatic software to Create a novel aptamer library which undergoes a microarray screening process. Sequence information and, if available, folding information (optional) from a SELEX experiment is used as input for the resample software. Resample outputs a novel focused aptamer library that is small enough to fit on a DNA microarray. The microarray is incubated with a fluorescently labeled target before washing and scanning to measure the binding strength of all aptamers. Figure adapted from Kinghorn et al. [118].

In 2016 Kinghorn et al. reported a novel strategy of aptamer affinity maturation by library resampling from SELEX sequence data [118]. This approach relies on the principle that classical SELEX is unlikely to select the best possible aptamer, but is highly likely to select family members of the best possible aptamer. The sequence of the best possible aptamer is hidden within the sequences of its family members. To make use of this aptamer family information, the authors coded the bioinformatic software “Resample”, which uses information from a SELEX experiment in terms of an aptamer family motif and any available folding information. This information is used to generate a novel library that consists of every possible aptamer permutation within the aptamer family. This library is focused on a particular area of sequence space, representing it thoroughly while still having a library size small enough to fit onto a DNA microarray for screening (Figure 6). To demonstrate this process, the sequence data from a previous selection against the malarial antigen *Plasmodium falciparum* lactate dehydrogenase (PfLDH) was input into Resample to generate a library of 186,624 novel aptamer sequences within the specified aptamer family. This library was ordered on a DNA microarray that was incubated with 50 nM Alexa Fluor 555 labeled PfLDH (target) and 1 µM Alexa Fluor 647 labeled human lactate dehydrogenase B (counter-target) and washed and scanned to measure both binding affinity and binding specificity for all aptamers. The lead candidates were further characterized using microscale thermophoresis to show an order of magnitude improvement in binding affinity *K*_D_ value. The authors provide Resample as a hosted web service (website available: http://resample.azurewebsites.net) and state that affinity maturation using Resample round should just take two days, excluding microarray shipping time. While many microarray aptamer optimization processes use mutation and recombination, taking many small evolutionary steps to arrive at an optimum, Resample takes all possible selection sequence information and takes a single evolutionary leap to an optimum. In this way, a single Resample diversification round can be used to hone in on the sequence space containing the best possible aptamer sequence [118].

## 7. Conclusions and Future Perspectives

Simulation of aptamer selection has given insight into the SELEX process, including the optimum protein/target concentration, the detrimental effect of low relative partitioning efficiency, the effect of background binding, and the stochastic nature of SELEX. The area least accurately modeled in SELEX simulations is aptamer binding. Development of more accurate aptamer binding models and applying them in simulation may lead to new insights into SELEX.

In silico approaches have been used to classify molecular interactions between binding macromolecules into discrete categories. Each categorized interaction is assigned a probability and level of importance, which is translated into a score. By grouping all the scores, it is possible to distinguish a poor binding interaction from a strong interaction. Most programs have been developed for protein–protein interactions, but in recent years useful software has been devised to investigate DNA–target interactions. These in silico tools for both whole aptamer and fragment approaches have aided aptamer scientists in improving their selections and identifying novel high-affinity aptamers.

It is commonly known that the huge number of candidates in a random nucleic acid library cannot be covered by a single SELEX experiment. One of the solutions to reduce that number or increase the coverage of selection is to introduce patterns into the library. In silico methods include defining alternating purine and pyrimidine patterns leading to the increase in occurrence of stem–loop structures or more complicated structures such as quadruplexes. The design could also be combined with in vitro experiments to access the structural diversity of certain patterned libraries by CD and NMR. Such approaches will effectively help to increase the success rate of identifying active binders in the selection process.

HTS technology shows high potential to replace the cloning and Sanger sequencing methods applied in traditional SELEX. By integrating an HTS step into SELEX, researchers can successfully reduce the selection rounds and the need for post-selection experiments to identify optimal aptamer sequences. These advantages of HTS technology encourage the rapid development of aptamer-based bioinformatic tools. There are several software packages and databases customized for aptamer scientists (Table 2) to analyze the large amount of HTS data based on different strategies. It will be useful to consider how best to compare these tools, using the same batch of data with multiple validations from different research groups.

In silico aptamer optimization has not been widely adopted. This may be due to the observation that most studies either achieve only mild affinity improvements or that the optimization method, while successful, is prohibitively expensive. Nonaka et al. achieved binding improvement of an order of magnitude by using a low-cost method, albeit labor-intensive in SPR measurements [117]. Kinghorn et al. achieved binding improvement of an order of magnitude with a low-cost method that can be performed in two days, excluding microarray shipping time [118]. Many aptamer optimization studies are stand-alones without follow-up or verification by other research groups. For the aptamer community to adopt in silico aptamer optimization, replicate studies need to be performed to strengthen and further validate in silico aptamer optimization methods.

Bioinformatic approaches have been used to improve both aptamers and their selection. In this review we have outlined a broad range of aptamer bioinformatics techniques including simulation of aptamer selection, aptamer selection by molecular dynamics, patterning of libraries, identification of lead aptamers from HTS data, and in silico aptamer optimization. Aptamers are particularly suited to bioinformatic techniques and their development and use can benefit aptamer scientific community.

## Figures and Tables

**Table 1:** Library design used by Martin et al. [64].

Pattern	Library Design
1	(RY)_3_-N_4_-(RY)_4_-N_3_-(RY)_4_-N_4_-(RY)_4_-N_3_-(RY)_3_
2	(RRYY)_2_-N_4_-(RRYY)-N_3_-(RRYY)-N_4_-(RRYY)-N_3_-(RRYY)-N_4_-(RRYY)_2_
3	(RRYY)_2_-N_4_-(RRRYYY)-N_4_-(RRRYYY)-N_4_-(RRRYYY)-N_4_-(RRYY)_2_
4	(RRYY)_2_-N_4_-(RY)_3_-N_4_-(RY)_3_-N_4_-(RY)_3_-N_4_-(RRYY)_2_

^1^ Library designs of different patterns of alternating purine and pyrimidine. Pattern 1 library theoretically has 1.8 × 10^19^ sequences and Pattern 2 has 3 × 10^20^. Pattern 3 has three consecutive purines or pyrimidines, which may allow the formation of quadruplex, while Pattern 4 only allows alternating purines and pyrimidines. R is purine, Y is pyrimidine, and N is a random mixture of purine and pyrimidine. Table adapted from [64].

**Table 2:** Major open-source programs for SELEX (Systematic Evolution of Ligands by Exponential Enrichment) HTS (high-throughput sequencing) data analysis.

Program	Operation System	Language	Clustering Method	Validation Experiment
FASTAptamer	Mac/Linux	Perl	Levenshtein distance	HIV-1 Reverse Transcriptase
AptaCluster/AptaGUI	Mac/Linux/PC	Java	LSH and k-mer counting	IL-10RA
APTANI	Linux	Python	Structure motif-based clustering	Murine IL4Ra
AptaTrace	Mac/Linux/PC	C++, Java	Structure motif-based clustering	C-C chemokine receptor
PATTERNITY-seq	No details	No details	Levenshtein distance	Annexin-A2
